# Long-term effects of alcohol consumption on cognitive function in seniors: a cohort study in China

**DOI:** 10.1186/s12877-021-02606-y

**Published:** 2021-12-15

**Authors:** Lizhen Han, Jinzhu Jia

**Affiliations:** Department of Biostatistics, School of Public Health, Peking University, Beijing, China, No. 38, Xueyuan Road, Beijing, 100191 Haidian District China

**Keywords:** Alcohol consumption, Cognitive function, Seniors, Dynamic cox model

## Abstract

**Background:**

In the context of increasing global aging, the long-term effects of alcohol consumption on cognitive function in older adults were analyzed in order to provide rationalized health recommendations to the elderly population.

**Methods:**

The study used the Chinese Longitudinal Healthy Longevity Survey (CLHLS) dataset, from which 5354 Chinese seniors aged 65–112 years were selected as the subjects, spanning the years 1998–2018. Data on alcohol, diet, activity, and cognition were collected by questionnaire and cognitive levels were judged by the Mini-Mental State Examination scale (also referenced to the Functional Assessment Staging Test). Data cleaning and preprocessing was implemented by R software. The dynamic Cox model was applied for model construction and data analysis.

**Results:**

The results of the dynamic Cox model suggested that seniors who drank alcohol were at higher risk of cognitive decline compared to those who never drank (*HR =* 1.291, 95%*CI*: 1.175–1.419). The risk was similarly exacerbated by perennial drinking habits (i.e., longer drinking years, *HR =* 1.008, 95%*CI*: 1.004–1.013). Compared to non-alcoholic beverages, liquor (≥ 38°), liquor (< 38°), wine and rice wine all showed negative effects. Whereas, the risk of cognitive decline was relatively lower in seniors who consumed liquors (< 38°) and rice wine compared to the high-level liquor (*HR*: 0.672 (0.508, 0.887) and 0.732 (0.559, 0.957), respectively).

**Conclusions:**

Alcohol consumption has a negative and long-term effects on cognitive function in seniors. For the elderly, we suggested that alcohol intake should be avoided as much as possible.

**Supplementary Information:**

The online version contains supplementary material available at 10.1186/s12877-021-02606-y.

## Introduction

Cognitive issues can no longer be ignored. In 2019, more than 50 million people worldwide suffered from dementia, with an average of one new case of dementia every 3 seconds, and by 2030 the number will reach a frightening 75 million [1, 2]. Dementia not only jeopardizes the cognitive health of individuals (memory, executive function, attention, etc. [3]), but also imposes a huge burden on society. Previous estimates showed that the global health care cost of dementia has already reached $1 trillion in 2019, and the cost is expected to double again by 2030 [2, 4].

China will enter the moderate aging society (i.e., a society in which more than 14% of the population is 65 years or older) during the “14th Five Year Plan” period (2021–2025), with the number of elderly people exceeding 300 million and the number of people with dementia reaching 16.6 million by 2030 [5–7]. By that time, China will face even more severe challenges and tests.

The development of cognitive function depends on numerous influencing factors. In addition to the more general demographic characteristics (e.g., age, gender, and education), factors such as diet, exercise, disease, and genetics are also covered [8–10]. The effect of alcohol on cognition has been controversial. Some studies have suggested a potential beneficial effect of low or moderate alcohol intake on cognitive function [11–14], while others have shown no or opposite effects of moderate alcohol consumption on cognitive status [15–17]. There is still much debate on this issue and no definitive conclusions can be drawn at this time. However, the harmful effects of excessive alcohol consumption on cognitive function have been well documented [18–21].

Based on the above, more evidence needs to be supplemented. The purpose of this study was to provide more targeted suggestions for the seniors by analyzing the long-term effects of alcohol consumption habits on individual cognitive function of the elderly in China, and to provide additional evidence for the implementation of relevant health promotion measures.

## Methods

### Data sources

**Fig. 1:**
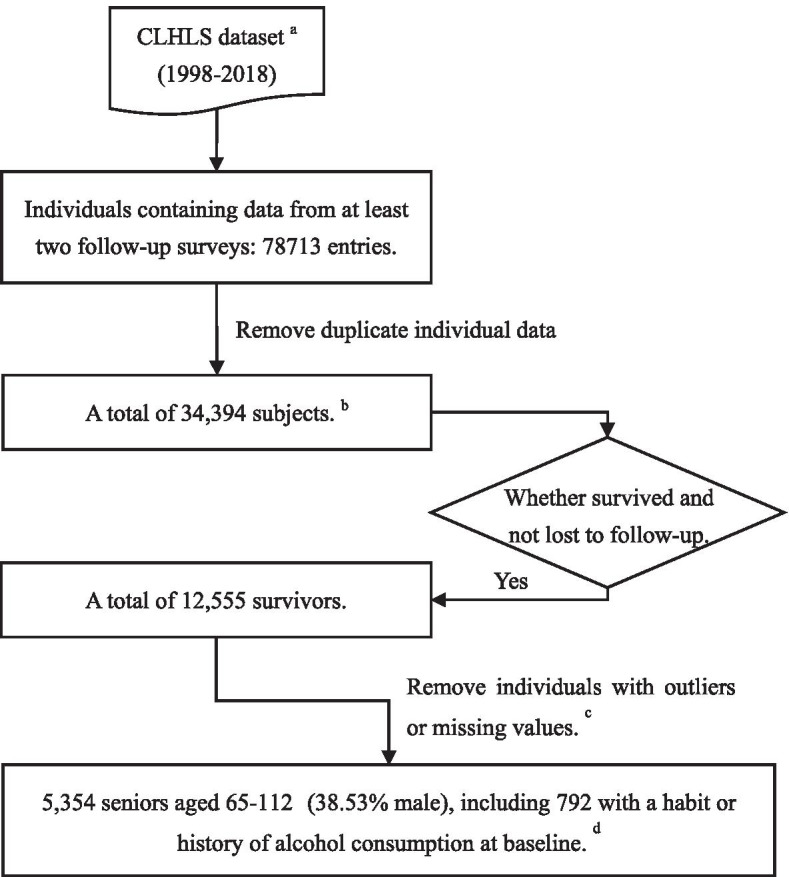
Flow chart of the sample screening process. ^a^ The time points covered by this dataset contain the following years: 1998, 2000, 2002, 2005, 2008, 2011, 2014 and 2018. ^b^ The number includes individuals from the first baseline survey in 1998 and new participants added in follow-up surveys. ^c^ Variables for cognition, alcohol consumption, diet, and activity were included (details were shown in Additional file 1). Among them, individuals with cognitive-related MMSE scores exceeding the range of − 3 < MMSE z-score < 3 would be excluded (after adjustment for gender and age). ^d^ Age was based on their records at the time of the baseline survey

The sample came from the Chinese Longitudinal Healthy Longevity Study (CLHLS), covering a total of eight surveys from 1998 to 2018 [22, 23]. This survey collected data in the form of a questionnaire on numerous aspects (e.g., sociodemographic characteristics, health, lifestyle, etc.). In this study, we selected seniors who participated in three consecutive surveys (including their baseline surveys) from a total of six data sets, and the time periods they covered were 1998–2002 (specific survey years: 1998, 2000, 2002), 2000–2005 (2000, 2002, 2005), 2002–2008 (2002, 2005, 2008), 2005–2011 (2005, 2008, 2011), 2008–2014 (2008, 2011, 2014) and 2011–2018 (2011, 2014, 2018). A total of 5354 adults aged 65 years or older (38.53% male) were included after stratified screening, covering 22 of the 34 provinces in China (*n*_*urban*_ = 3,175 (59.30%)). Among them, 2867 had a follow-up duration of 7 years, and 828 and 1659 participants had a duration of 5 and 6 years, respectively. In addition, 792 had a history or habit of alcohol consumption at their baseline survey. The sample screening process was shown in Fig. 1.

### Alcohol consumption data

The drinking data were divided into four main parts. The first part recorded whether individuals drink alcohol or not, including three categories of long-term drinking, abstinent and never drinking, which were categorical variables: never drinking was recorded as “0” and the rest were indicated by “1”. The remaining three sections focus on the drinking population, including the number of years of drinking, type of alcohol (representing the category most commonly consumed by individuals, the specific alcohol by volume of each type was presented in Additional file 1) and daily consumption (unit: tael/day, 1 tael = 50 ml). The liquor involved in this study mainly refer to Chinese baijiu.

### Other data

Data related to cognition, such as demographic characteristics, family, diet, activity, and disease, were analyzed as covariates. Among them, “1” and “2” in the gender variable represented males and females, respectively. The values of the diet component variables (e.g., vegetable, meat, sugar, etc.) were categorized into three levels, representing the frequency of food intake, from low to high as “frequently”, “occasionally” and “rarely or never”. The activity component (except for “exercise”: “0” for never exercising and “1” for the opposite) was also graded in the same way, indicating the frequency of activity participation. It mainly covers categories such as outdoor activities, reading, housework, etc. Due to the constraints of the model, the variables of the diet and activity sections were analyzed as quantitative variables (larger values represent lower frequencies). In addition, the residence information reveals the residence status of the elderly, which was divided into three categories: living with family, living alone and living in a rest home. Detailed information was shown in the Additional file 1.

### Judging criteria for cognitive function

The Mini-Mental State Examination (MMSE) scale was used to assess the cognitive status of each individual in CLHLS, and the score was utilized as a basis for evaluating the cognitive level (out of 30 points) [24]. Based on the results of the study conducted by Xin Ying Chua et al. on this scale [25], our study classified individuals’ cognitive levels into six levels (corresponding to stages 1–7 of the Functional Assessment Staging Test (FAST), where stages 1 and 2 were combined into one level), representing different degrees of cognitive status: from low to high corresponding to health, mild cognitive impairment, mild dementia, moderately dementia, moderately severe dementia and severe dementia in the FAST, respectively [26]. The MMSE cut-off points for each stage were 24, 21, 15, 12, and 5 for subjects with primary education (≤ 6 years of education) and 26, 25, 17, 15, and 10 for those with secondary education (> 6 years of education).

### Statistics analysis

R 4.0.4 was applied to data analysis and processing, and data modeling was implemented using the dynamic Cox model. A probability level of *p* < 0.05 represented the result with statistical significance.

### Dynamic cox model

Dynamic Cox models were used in this study to analyze the long-term effects of drinking habits on the cognitive status of older adults. It is an extension of the Cox proportional hazards regression model: the introduction of time-dependent covariates allows for a better analysis of the impact on outcomes due to changes in factors over time [27]. The formula for the dynamic Cox model is as follows:

$$\lambda \left(t|X\right)={\lambda}_0(t)\mathit{\exp}\left\{\sum_{i=1}^n{\beta}_i{X}_i(t)\right\}$$

where *t* represents the survival time, *λ*(*t*| *X*) is the hazard function at moment *t* determined by a set of n covariates, *λ*_0_(*t*) is the baseline hazard function and *β* is the regression coefficient of the time-dependent covariate *X*(*t*).

In this study, we used the change in individual cognitive function as the outcome variable: if the FAST stage increased compared to the previous survey, a “1” was recorded, representing a decrease in the individual’s cognitive level, and vice versa, a “0” was recorded. According to the time interval of the subjects, the data set was then processed using the “tmerge” function (from the “survival” package of R software) to recode the time-dependent covariates and generate a new data set. The survival time of each individual was obtained by calculating the time difference among the year and month data of each survey.

Four models were constructed to analyze the relationship between alcohol consumption and cognitive status in older adults. For all older adults, the analysis was first conducted using a univariate time-dependent model with the following model:

- **Model I:** Surv (tstart, tstop, status) ~ drinking or not

where tstart and tstop represent the start and end times of each survey period (survival time), and status represents the change in cognitive status.

Further, all covariates were selected using stepwise regression (Akaike information criterion (AIC), “both” direction) to form the time-dependent model II (30 in total, with 20 variables included, listed in Additional file 2) based on the exclusion of multicollinearity.

- **Model II:** Surv (tstart, tstop, status) ~ drink + age + gender + years of education + residence + marital status + vegetable + meat + fish + bean + pickle + sugar + garlic + exercise + outdoor activities + read + raise pet + mahjong + tv/radio + community activities

For those with a drinking habit or history of drinking at the time of the baseline survey (i.e., long-term drinkers and abstainers), we performed the analysis again using the same methods described above and constructed two time-dependent models, which were presented below.

- **Model III:** Surv (tstart, tstop, status) ~ drinking years + type of alcohol + drinking volume
- **Model IV:** Surv (tstart, tstop, status) ~ drinking years + type of alcohol + drinking volume + age + gender + years of education + smoking or not + fruit + vegetable + fish + bean + sugar + housework + outdoor activities + gardening + raise pet + mahjong

In order to conduct a more comprehensive analysis for comparison while avoiding the drawbacks of unbalanced data distribution, we randomly selected 500 individuals (approximating the number of drinkers in the main alcohol categories) from the non-drinking population (*n* = 4562) and repeated the sampling 10 times. The sample taken each time was also combined with the drinking population and applied to Model IV.

## Results

### Basic characteristic

In this study, a total of 5354 older adults aged 65–112 years (baseline age) were selected as subjects. The mean age was 79.94 (9.85) years, with a ratio of 1:1.67 between older persons and the oldest old (with 80 years as the cut-off point [28]). Their mean MMSE scores for the three surveys were 27.07, 26.28, and 25.07, respectively, while the mean FAST stages were 2.28, 2.43, and 2.67, respectively. During the follow-up period, there were 2399 outcome events (20.30% were drinkers), with a much higher proportion of outcome events occurring in drinkers than in non-drinkers at their second follow-up (32.20% vs. 23.89%). Detailed information is presented in Additional file 3. In addition, 14.79% of seniors had alcohol consumption habits at the time of the baseline survey. Their mean age was 85.98 (6.69) years, and their mean years of drinking and mean daily citation were 51.73 (22.13) years and 2.96 (3.33) tael/day, respectively. Besides, liquor (≥ 38°) was the most commonly consumed type among them, accounting for about 50%.

### Dynamic cox model

**Fig. 2:**
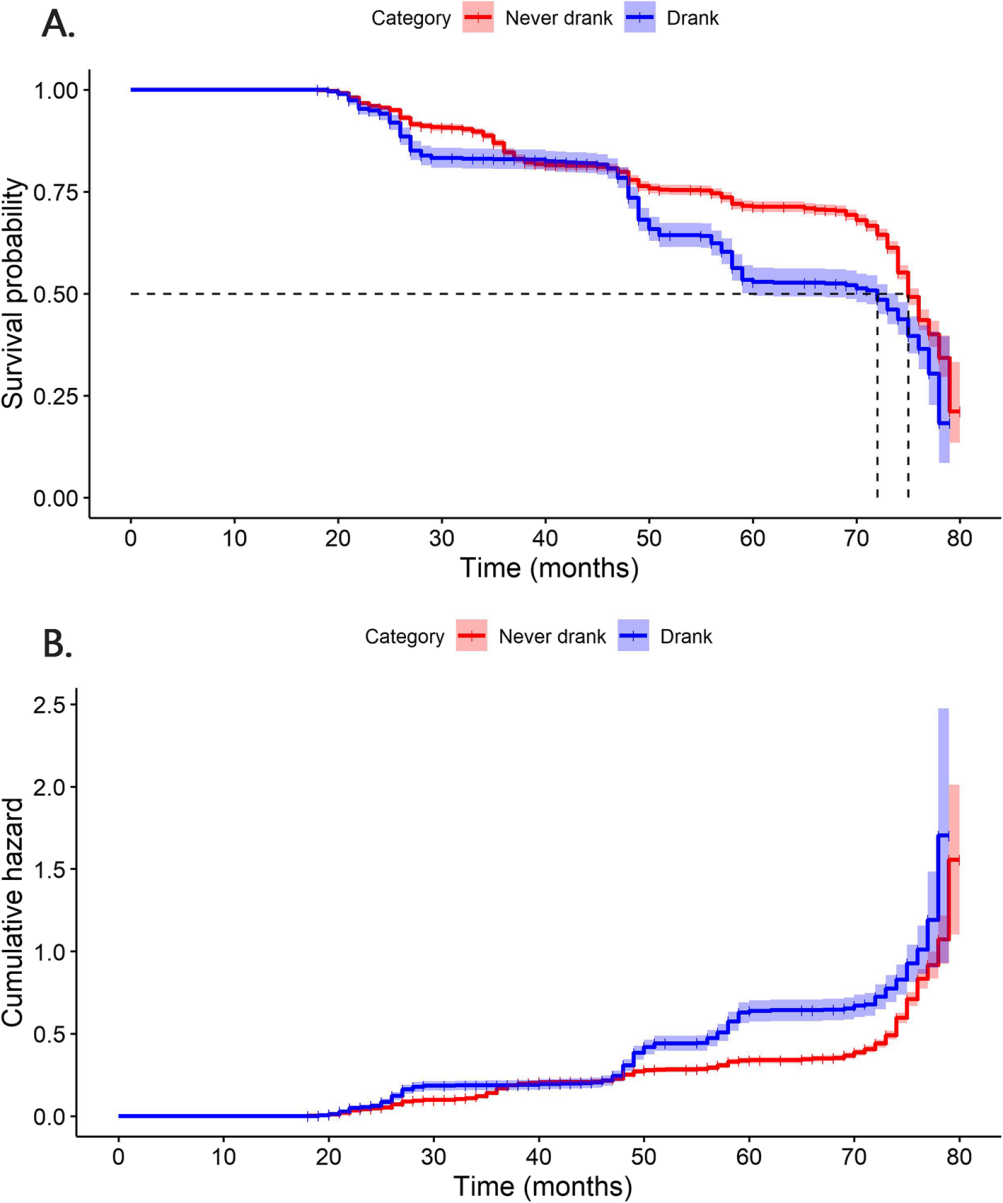
Survival plots for seniors who never drank and those who drank: survival probability (**A**) and cumulative hazard (**B**)

We plotted Kaplan-Meier (KM) curves and cumulative hazard curves for the “Drink” variable and constructed Model I (concordance = 0.53, logrank test = 52.92, *p* < 0.000) simultaneously. The results of the univariate analysis showed that seniors who had a drinking habit were at greater risk for substantial decline in cognitive function over time than those who never drank alcohol. In addition, the median survival time of the drinking population was slightly earlier than that of the elderly who had never consumed alcohol. The results were shown in Fig. 2 and Table 1.

**Table 1:** Results of dynamic Cox model I (*n* = 5354)

	coef	se (coef)	robust se	*z*	*p*	*HR*	95%*CI* for *HR*	
							Lower bound	Upper bound
Drink	0.370	0.051	0.049	7.610	< 0.000	1.447	1.316	1.592

After adjusting for numerous covariates, the results of Model II (concordance = 0.69, logrank test = 1151, *p* < 0.000) still suggested that alcohol consumption increases the risk of cognitive impairment in older adults (hazard ratio [*HR*] = 1.291, 95% confidence interval [*CI*]: 1.175–1.419). Moreover, advanced age, being female, living in the rest home, widower/widow, frequent intake of vegetables (veg) and sugary foods, and regular participation in community activities showed the same negative effects. In contrast, longer years of education, trial separation, higher intake of meat, fish, legumes and garlic, more participation in outdoor activities, reading, recreational activities such as mahjong and TV/radio reduced this hazard to some extent. The results were shown in Table 2.

**Table 2:** Results of dynamic Cox model II (n = 5354)

Factor	coef	se (coef)	robust se	*z*	*p*	*HR*	95%*CI* for *HR*	
							Lower bound	Upper bound
Age	0.049	0.002	0.002	23.761	0.000	1.050	1.046	1.055
Gender	0.128	0.053	0.047	2.750	0.006	1.138	1.038	1.246
Years of education	−0.021	0.009	0.008	−2.519	0.012	0.980	0.964	0.996
Drink	0.255	0.054	0.048	5.302	0.000	1.291	1.175	1.419
Residence								
Family	Reference							
Alone	−0.086	0.061	0.056	−1.550	0.121	0.918	0.823	1.023
Rest home	0.380	0.113	0.096	3.942	0.000	1.462	1.210	1.766
Marital status								
Married	Reference							
Separated	−0.389	0.197	0.193	−2.018	0.044	0.678	0.464	0.989
Divorced	0.557	0.320	0.301	1.853	0.064	1.745	0.968	3.146
Widowed	0.166	0.056	0.051	3.266	0.001	1.181	1.069	1.305
Never married	0.325	0.215	0.202	1.609	0.108	1.384	0.932	2.056
Diet								
Veg	−0.098	0.028	0.027	−3.609	0.000	0.907	0.860	0.956
Meat	0.062	0.032	0.030	2.078	0.038	1.064	1.004	1.127
Fish	0.061	0.033	0.030	2.007	0.045	1.063	1.001	1.128
Legume	0.079	0.031	0.027	2.765	0.006	1.082	1.023	1.145
Pickle	0.037	0.026	0.024	1.530	0.126	1.037	0.990	1.087
Sugar	−0.108	0.027	0.025	−4.246	0.000	0.898	0.854	0.943
Garlic	0.107	0.029	0.027	3.946	0.000	1.113	1.055	1.173
Activity								
Exercise	−0.089	0.045	0.041	−2.215	0.027	0.914	0.844	0.990
Outdoor	0.055	0.026	0.025	2.234	0.025	1.057	1.007	1.109
Read	0.084	0.045	0.041	2.056	0.040	1.088	1.004	1.179
Raise pet	−0.040	0.026	0.025	−1.591	0.112	0.961	0.915	1.009
Mahjong	0.131	0.042	0.039	3.316	0.000	1.140	1.055	1.231
TV/radio	0.097	0.028	0.026	3.704	0.000	1.102	1.047	1.160
Community	−0.091	0.044	0.041	−2.206	0.027	0.913	0.843	0.990

Further modeling analysis of drinking-related variables was conducted for those who had a habit or history of drinking at baseline. Model III (concordance = 0.57, logrank test = 31.31, *p* < 0.000) showed that longer years of drinking tended to expand the hazard of cognitive decline (*HR* = 1.008, 95%*CI:* 1.004–1.013), while low-alcohol liquor (< 38°) demonstrated a positive effect (*HR* = 0.672, 95%*CI:* 0.508–0.887) compared to the consumption of strong liquor (≥ 38°). The results were presented in Table 3. The same conclusions were drawn from model IV (concordance = 0.67, logrank test = 145.9, *p* < 0.000) after adjusting for other covariates (Table 4). Meanwhile, we also found that this risk was lower in seniors who consumed rice wine (*HR* = 0.732, 95%*CI:* 0.559–0.957). Older drinkers who were active in mahjong-type activities and who regularly consumed fish and legumes had a lower risk of rapid deterioration in cognitive levels. In addition to this, the negative effects of age, female, and high frequency intake of vegetables and sugary foods were once again confirmed.

**Table 3:** Results of dynamic Cox model III (*n* = 792)

Factor	coef	se (coef)	robust se	*z*	*p*	*HR*	95%*CI* for *HR*	
							Lower bound	Upper bound
Drinking years	0.008	0.002	0.002	3.654	0.000	1.008	1.004	1.013
Category								
Liquor (≥ 38°)	Reference							
Liquor (< 38°)	−0.398	0.155	0.142	−2.805	0.005	0.672	0.508	0.887
Wine	0.201	0.167	0.155	1.297	0.195	1.223	0.902	1.658
Rice wine	−0.165	0.133	0.133	−1.243	0.214	0.848	0.653	1.100
Beer	−0.740	0.427	0.421	−1.759	0.079	0.477	0.209	1.088
Others	−0.751	0.454	0.408	−1.839	0.066	0.472	0.212	1.051
Drinking volume	−0.031	0.020	0.021	−1.461	0.144	0.970	0.929	1.011

**Table 4:** Results of dynamic Cox model IV (n = 792)

Factor	coef	se (coef)	robust se	*z*	*p*	*HR*	95%*CI* for *HR*	
							Lower bound	Upper bound
Age	0.031	0.008	0.007	4.745	0.000	1.031	1.018	1.045
Gender	0.510	0.131	0.115	4.441	0.000	1.665	1.329	2.084
Years of education	−0.005	0.015	0.014	−0.342	0.732	0.995	0.970	1.023
Drinking years	0.006	0.002	0.002	2.717	0.007	1.006	1.002	1.010
Category								
Liquor (≥ 38°)	Reference							
Liquor (< 38°)	−0.332	0.157	0.141	−2.358	0.018	0.719	0.545	0.946
Wine	−0.034	0.173	0.161	−0.214	0.831	0.966	0.705	1.325
Rice wine	−0.313	0.139	0.137	−2.283	0.022	0.732	0.559	0.957
Beer	−0.718	0.428	0.431	−1.666	0.096	0.488	0.210	1.135
Others	−0.609	0.458	0.398	− 1.529	0.126	0.544	0.249	1.187
Drinking volume	−0.001	0.019	0.017	−0.030	0.976	0.999	0.967	1.034
Smoke	0.179	0.124	0.112	1.599	0.110	1.197	0.960	1.491
Diet								
Fruit	−0.094	0.056	0.052	−1.793	0.073	0.910	0.821	1.009
Veg	−0.185	0.069	0.068	−2.738	0.006	0.831	0.728	0.949
Fish	0.241	0.080	0.077	3.112	0.002	1.272	1.093	1.481
Legume	0.252	0.076	0.078	3.215	0.001	1.287	1.104	1.501
Sugar	−0.171	0.068	0.064	−2.651	0.008	0.843	0.743	0.956
Activity								
Housework	0.098	0.063	0.061	1.608	0.107	1.103	0.979	1.243
Outdoor	0.109	0.063	0.065	1.687	0.092	1.116	0.982	1.267
Gardening	0.155	0.098	0.089	1.748	0.081	1.168	0.981	1.390
Raise pet	−0.105	0.069	0.066	−1.594	0.111	0.900	0.792	1.024
Mahjong	0.214	0.099	0.092	2.325	0.020	1.238	1.034	1.483

In addition, the results of the analysis conducted after sampling showed that the consumption of liquor (≥ 38°)and wine were high risk factors compared to non-alcoholic beverages (confirmed in all 10 analyses). Meanwhile, the negative effects of liquor (< 38°) and rice wine were also revealed (9 times). The results are detailed in Additional file 4 (Tables S1-S10).

**Fig. 3:**
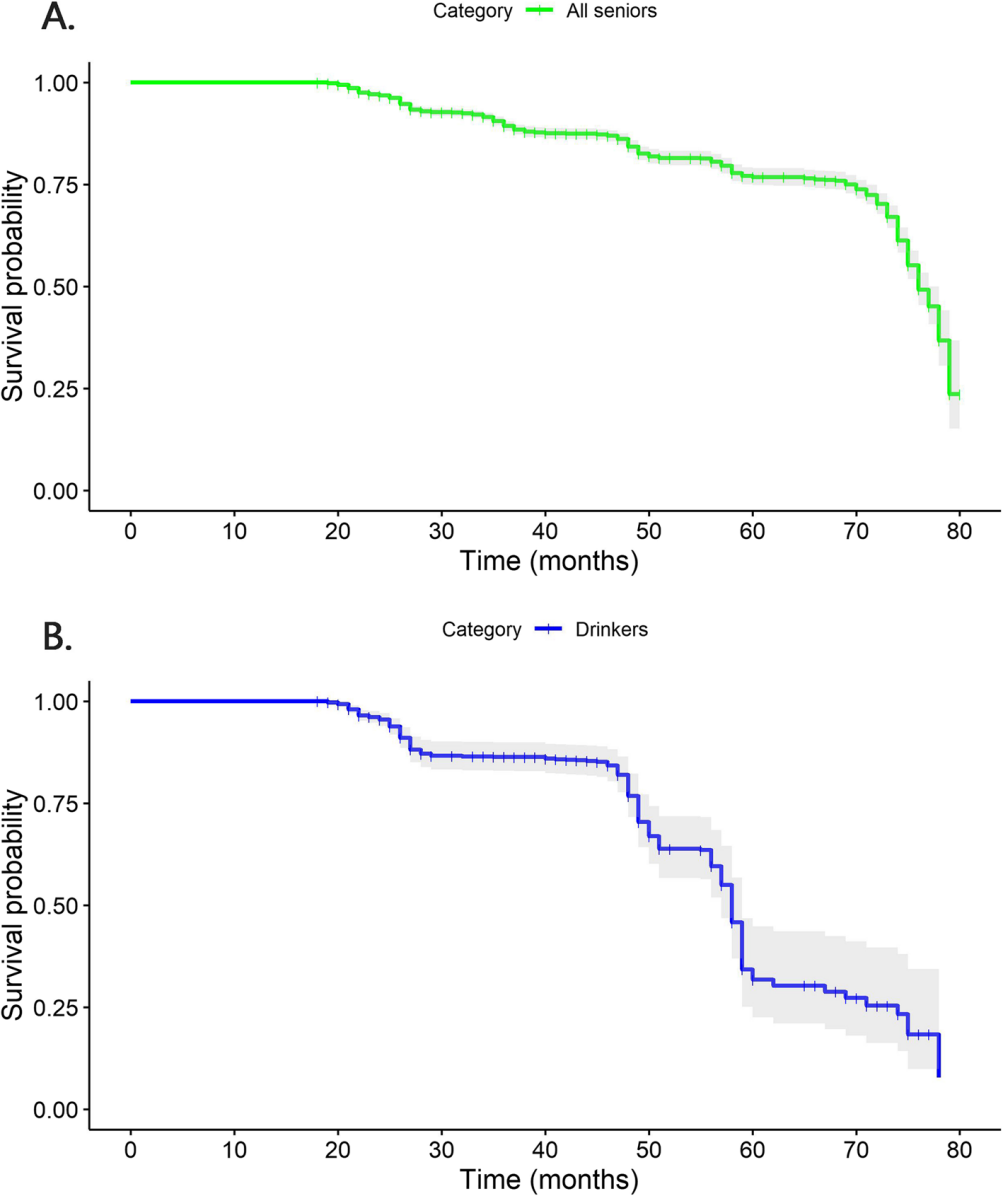
Survival plots (after adjustment) for all elderly (**A**) and elderly drinkers at baseline survey (**B**)

Moreover, by comparing the survival curves of model II and model IV (Fig. 3), it was found that the long-term effects were more significant and the curves dropped more dramatically over time for the elderly drinkers.

## Discussion

After modeling analysis, we explored the long-term effects of alcohol on cognitive function in an elderly Chinese population, and the results confirmed the negative effects of alcohol consumption. This is consistent with the findings of many previous studies [17, 29–32]. Older adults with alcohol consumption habits had a faster rate of cognitive decline over time and were more likely to experience cognitive impairment. This gap was most pronounced in the period of 4–6 years (mean age: 87.23 [6.18]) after the baseline survey. Alcohol affects cognitive function primarily through its neurotoxic effects, which may be mediated directly through damage to brain structures or indirectly through malnutrition, metabolite toxicity, electrolyte imbalance, or adverse physiological disorders including liver disease and infection [33]. Earlier studies also confirmed that even moderate doses impaired performance by attenuating the brain’s ability to detect action slips [34].

Notably, high-level liquor, low-level liquor, wine and rice wine all had a higher risk of cognitive impairment compared to non-alcoholic beverages. The behavioral consequences of ethanol’s actions on brain neurochemistry, as well as the neurochemical effects themselves, are highly dose- and time-dependent [35]. High levels of ethanol intake can further exacerbate the risk of developing cognitive impairment and dementia [36, 37]. Among the many types of alcohol, low-level liquor and rice wine showed a more positive effect compared to the strong spirit. However, the causes and specific mechanisms of action as to why lower risks were presented only in low-level liquors and rice wine, and why they differ from other types of alcohol are not clear and need further study. Initial speculation is that it may be related to their unique raw materials and brewing processes. Moreover, our study did not find a protective effect of moderate alcohol consumption on cognition in seniors [14]. Phenomena such as mixed drinking of multiple types of alcohol may have masked the true effects. Similarly, individuals with a unique drinking culture in China (liquor-dominated) will consume higher levels of alcohol than drinkers of other alcohol types. The study by Marinus N. Verbaten confirmed a U-shaped association between alcohol consumption and cognitive functioning in elderly low to moderate drinkers (aged > 65 years) [38], and this nonlinear association may also have played a confounding role. Nevertheless, due to the lack of specific quantitative drinking data, we were not able to give accurate inferences. It is worth affirming, however, that high levels of alcohol consumption must be harmful [18, 19].

An in-depth profile of the drinking population found that longer years of drinking tended to be associated with a higher risk of cognitive impairment, revealing persistent cognitive impairment caused by long-term alcohol consumption. This also explained to some extent the need for timely abstinence from alcohol. As stated above, the time dependence of ethanol may be the main factor. Meanwhile, long-term heavy alcohol consumption can lead to thiamine deficiency and inadequate nutritional intake, which in turn can predispose to the development of Wernicke-Korsakoff syndrome (long-term persistent amnesia and other symptoms) [39]. Furthermore, the available studies showed that people who consume alcohol tend to have more health problems than non-drinkers, such as high blood pressure, diabetes, ulcer disease, deterioration of walking function and exercise capacity [30, 40–42]. Compared to the limited benefits, we do not recommend the consumption of alcoholic products. In regards to the average daily consumption, no association between it and the cognitive level of the elderly was found in this study. There may be a non-linear association [36, 37, 43, 44], the details of which also need to be explored in depth.

In addition, we also found some other valuable results from the covariate-adjusted model. This covers not only the proven beneficial (longer years of education, higher intake of fish, legumes and garlic, more participation in outdoor activities, etc.) and detrimental (e.g. age, being female, living in the rest home, widower/widow, frequent intake of sugary foods) factors [8, 9, 45, 46], but also individual factors that contradict the previous findings [9]. The frequency of vegetable/meat intake, for example, has been advocated by many as a dietary pattern with more vegetables and less animal fat, but the current study in the Chinese elderly population came to the opposite conclusion. This is an interesting finding, although the exact reasons cannot be explained yet. We recommend that more comprehensive comparative studies should be conducted for different populations in order to make more accurate judgments about such issues.

This study comprehensively explored the long-term effects of drinking habits through survival analysis between alcohol consumption and cognitive levels of older adults. However, we were unable to obtain information on individual-specific alcohol intake, which made it difficult to quantify the effect of alcohol intake. Problems such as the effects of simultaneous consumption of multiple types of alcohol and the accuracy of self-reported consumption were similarly elusive. Coupled with the fact that the context of alcohol and culture varies from country to country, further research evidence on this issue needs to be added.

## Conclusion

Alcohol consumption has a negative and long-term effects on cognitive function in seniors, and the risk increases the longer the year of consumption. For the Chinese elderly population in a liquor (baijiu) culture, this study suggested that alcohol intake should be avoided as much as possible. In addition, avid high alcohol drinkers can choose low-level liquor or rice wine instead of strong liquor and gradually abstain from alcohol to reduce the risk of cognitive impairment.

## Supplementary Information

**Additional file 1.** List of variables explanation.

**Additional file 2.** List of variables incorporated in the variable selection process.

**Additional file 3.** Basic information for participants.

**Additional file 4: Table S1** Results of dynamic Cox model IV (contains samples from the 1st random sample, *n* = 1292).

## Data Availability

The datasets generated and/or analysed during the current study are available in the Peking University Open Research Data repository. https://opendata.pku.edu.cn/dataset.xhtml?persistentId=doi%3A10.18170%2FDVN%2FWBO7LK

## References

[1] WHO (2016). World report on ageing and health.

[2] International AsD (2019). World Alzheimer report 2019: attitudes to dementia.

[3] International Classification of Diseases, Eleventh Revision (ICD-11) [https://icd.who.int/browse11/l-m/en#/http://id.who.int/icd/entity/546689346].

[4] Wimo A, Guerchet M, Ali GC, Wu YT, Prina AM, Winblad B (2017). The worldwide costs of dementia 2015 and comparisons with 2010. Alzheimers Dement.

[5] Ministry of Civil Affairs press conference on October 23 [http://lyzx.mca.gov.cn:8280/asop/templates/n30/interviewlog.html].

[6] China Enters a Moderate Aging Society [https://spachina.com/2021/01/21/china-enters-a-moderate-aging-society/].

[7] International AsD (2015). World Alzheimer report 2015: the global impact of dementia. In.

[8] Wang J, Xiao LD, Wang K, Luo Y, Li XM. Gender differences in cognitive impairment among rural elderly in China. Int J Environ Res Public Health. 2020;17(10):3724. 10.3390/ijerph17103724 PMC7277614 32466167

[9] Wesselman LMP, van Lent DM, Schröder A, van de Rest O, Peters O, Menne F, et al. Dietary patterns are related to cognitive functioning in elderly enriched with individuals at increased risk for Alzheimer’s disease. Eur J Nutr. 2021;60(2):849–60. 10.1007/s00394-020-02257-6 PMC7900077 32472387

[10] Wu ZJ, Wang ZY, Hu BQ, Zhang XH, Zhang F, Wang HL (2020). Relationships of accelerometer-based measured objective physical activity and sedentary behaviour with cognitive function: a comparative cross-sectional study of China's elderly population. BMC Geriatr.

[11] Fischer K, van Lent DM, Wolfsgruber S, Weinhold L, Kleineidam L, Bickel H, et al. Prospective associations between single foods, Alzheimer's dementia and memory decline in the elderly. Nutrients. 2018;10(7):852. 10.3390/nu10070852 PMC6073331 29966314

[12] Panza F, Frisardi V, Seripa D, Logroscino G, Santamato A, Imbimbo BP (2012). Alcohol consumption in mild cognitive impairment and dementia: harmful or neuroprotective?. Int J Geriatr Psychiatry.

[13] Peters R, Peters J, Warner J, Beckett N, Bulpitt C (2008). Alcohol, dementia and cognitive decline in the elderly: a systematic review. Age Ageing.

[14] Rehm J, Hasan OSM, Black SE, Shield KD, Schwarzinger M. Alcohol use and dementia: a systematic scoping review. Alzheimers Res Ther. 2019;11:1. 10.1186/s13195-018-0453-0 PMC6320619 30611304

[15] Hogenkamp PS, Benedict C, Sjogren P, Kilander L, Lind L, Schioth HB (2014). Late-life alcohol consumption and cognitive function in elderly men. Age (Dordr).

[16] Yeung SLA, Leung GM, Chan WM, Hui YF, Lam TH, Schooling CM (2011). Moderate alcohol use and cognitive function in an elderly Chinese cohort. J Am Geriatr Soc.

[17] Jarvenpaa T, Rinne JO, Koskenvuo M, Raiha I, Kaprio J (2005). Binge drinking in midlife and dementia risk. Epidemiology.

[18] Lopes MA, Furtado EF, Ferrioli E, Litvoc J, Bottino CM (2010). Prevalence of alcohol-related problems in an elderly population and their association with cognitive impairment and dementia. Alcohol Clin Exp Res.

[19] Kim JW, Lee DY, Lee BC, Jung MH, Kim H, Choi YS (2012). Alcohol and cognition in the elderly: a review. Psychiatry Investig.

[20] Oslin DW, Cary MS (2003). Alcohol-related dementia: validation of diagnostic criteria. Am J Geriatr Psychiatry.

[21] Thomas VS, Rockwood KJ (2001). Alcohol abuse, cognitive impairment, and mortality among older people. J Am Geriatr Soc.

[22] Chinese Longitudinal Healthy Longevity Survey (CLHLS)-Duke University School of Medicine [https://sites.duke.edu/centerforaging/programs/chinese-longitudinal-healthy-longevity-survey-clhls/].

[23] Chinese Longitudinal Healthy Longevity Survey (CLHLS)-Peking University [https://opendata.pku.edu.cn/dataverse/CHADS].

[24] Folstein MF, Folstein SE, McHugh PR (1975). "mini-mental state". A practical method for grading the cognitive state of patients for the clinician. J Psychiatr Res.

[25] Chua XY, Choo RWM, Ha NHL, Cheong CY, Wee SL, Yap PLK (2019). Mapping modified mini-mental state examination (MMSE) scores to dementia stages in a multi-ethnic Asian population. Int Psychogeriatr.

[26] Sclan SG, Reisberg B (1992). Functional assessment staging (FAST) in Alzheimer's disease: reliability, validity, and ordinality. Int Psychogeriatr.

[27] Fisher LD, Lin DY (1999). Time-dependent covariates in the cox proportional-hazards regression model. Annu Rev Public Health.

[28] WHO (2000). Men ageing and health: achieving health across the life span.

[29] Zhou HD, Deng J, Li JC, Wang TJ, Zhang M, He HB (2003). Study of the relationship between cigarette smoking, alcohol drinking and cognitive impairment among elderly people in China. Age Ageing.

[30] Siddiquee AT, Kadota A, Fujiyoshi A, Miyagawa N, Saito Y, Suzuki H (2020). Alcohol consumption and cognitive function in elderly Japanese men. Alcohol.

[31] Futami SS, Ishikawa J, Jubishi C, Suzuki A, Morozumi A, Saito Y (2017). Prevalence and determinant of cognitive impairment in elderly patients with heart failure - a pilot study in a geriatric hospital. Eur J Heart Fail.

[32] Muhammad T, Govindu M, Srivastava S. Relationship between chewing tobacco, smoking, consuming alcohol and cognitive impairment among older adults in India: a cross-sectional study. BMC Geriatr. 2021;21(1):85. 10.1186/s12877-021-02027-x PMC7847155 33514331

[33] Neiman J (1998). Alcohol as a risk factor for brain damage: neurologic aspects. Alcohol-Clin Exper Res.

[34] Ridderinkhof KR, de Vlugt Y, Bramlage A, Spaan M, Elton M, Snel J (2002). Alcohol consumption impairs detection of performance errors in mediofrontal cortex. Science.

[35] Eckardt MJ, File SE, Gessa GL, Grant KA, Guerri C, Hoffman PL (1998). Effects of moderate alcohol consumption on the central nervous system. Alcohol Clin Exp Res.

[36] Zuccala G, Onder G, Pedone C, Cesari M, Landi F, Bernabei R (2001). Gruppo Italiano di Farmacoepidemiologia nell'Anziano I: dose-related impact of alcohol consumption on cognitive function in advanced age: results of a multicenter survey. Alcohol Clin Exp Res.

[37] Lao Y, Hou L, Li J, Hui X, Yan P, Yang K. Association between alcohol intake, mild cognitive impairment and progression to dementia: a dose–response meta-analysis. Aging Clin Exp Res. 2021;33(5):1175–85. 10.1007/s40520-020-01605-0 32488474

[38] Verbaten MN (2009). Chronic effects of low to moderate alcohol consumption on structural and functional properties of the brain: beneficial or not?. Hum Psychopharmacol.

[39] Martin PR, Singleton CK, Hiller-Sturmhofel S (2003). The role of thiamine deficiency in alcoholic brain disease. Alcohol Res Health.

[40] Mejldal A, Andersen K, Behrendt S, Bilberg R, Christensen AI, Lau CJ, et al. History of healthcare use and disease burden in older adults with different levels of alcohol use. A register-based cohort study. Alcohol Clin Exp Res. 2021;45(6):1237–48. 10.1111/acer.14615 33860951

[41] Nan X, Lu H, Wu J, Xue M, Qian Y, Wang W (2021). The interactive association between sodium intake, alcohol consumption and hypertension among elderly in northern China: a cross-sectional study. BMC Geriatr.

[42] Carvalho JKF, Pereira-Rufino LDS, Panfilio CE, Silva RDA, Cespedes IC (2021). Effect of chronic alcohol intake on motor functions on the elderly. Neurosci Lett.

[43] Gutwinski S, Schreiter S, Priller J, Henssler J, Wiers CE, Heinz A (2018). Drink and think: impact of alcohol on cognitive functions and dementia - evidence of dose-related effects. Pharmacopsychiatry.

[44] Beydoun MA, Beydoun HA, Gamaldo AA, Teel A, Zonderman AB, Wang Y (2014). Epidemiologic studies of modifiable factors associated with cognition and dementia: systematic review and meta-analysis. BMC Public Health.

[45] Kapusta J, Kidawa TM, Rynkowska-Kidawa M, IrzmaNski TR, Kowalski TJ (2020). Evaluation of frequency of occurrence of cognitive impairment in the course of arterial hypertension in an elderly population. Psychogeriatrics.

[46] Feng T, Feng Z, Jiang L, Yu Q, Liu K (2020). Associations of health behaviors, food preferences, and obesity patterns with the incidence of mild cognitive impairment in the middle-aged and elderly population: an 18-year cohort study. J Affect Disord.

