# Supplementary material for: Long-term effects of alcohol consumption on cognitive function in seniors: a cohort study in China
Source: BMC Geriatr. 2021 Dec 15;21:699. doi: 10.1186/s12877-021-02606-y (PMC8672616; doi:10.1186/s12877-021-02606-y)
Supplement: Supplementary file 4 — Additional file 4: Table S1 Results of dynamic Cox model IV (contains samples from the 1st random sample, n = 1292). [file 12877_2021_2606_MOESM4_ESM.docx]

| **Table.S1** Results of dynamic Cox model IV (contains samples from the 1^st^ random sample, n=1292) | | | | | | | | |
| --- | --- | --- | --- | --- | --- | --- | --- | --- |
| Factor | coef | se (coef) | robust se | *z* | *p* | *HR* | 95%*CI* for *HR* | |
|  |  |  |  |  |  |  | Lower bound | Upper bound |
| Age | 0.044 | 1.045 | 0.005 | 10.289 | 0.000 | 1.045 | 1.036 | 1.054 |
| Gender | 0.307 | 1.359 | 0.110 | 3.235 | 0.001 | 1.359 | 1.129 | 1.637 |
| Years of education | -0.010 | 0.990 | 0.013 | -0.790 | 0.429 | 0.990 | 0.967 | 1.015 |
| Drinking years | 0.004 | 1.004 | 0.002 | 1.943 | 0.052 | 1.004 | 1.000 | 1.007 |
| Category |  |  |  |  |  |  |  |  |
| Non-alcoholic | Reference | | | | | | | |
| Liquor (≥ 38º) | 0.676 | 1.965 | 0.161 | 4.622 | 0.000 | 1.965 | 1.476 | 2.618 |
| Liquor (< 38º) | 0.415 | 1.514 | 0.194 | 2.456 | 0.014 | 1.514 | 1.087 | 2.108 |
| Wine | 0.565 | 1.759 | 0.208 | 3.118 | 0.002 | 1.759 | 1.233 | 2.508 |
| Rice wine | 0.449 | 1.567 | 0.186 | 2.659 | 0.008 | 1.567 | 1.125 | 2.182 |
| Beer | 0.161 | 1.175 | 0.444 | 0.394 | 0.694 | 1.175 | 0.528 | 2.615 |
| Others | 0.158 | 1.171 | 0.470 | 0.392 | 0.695 | 1.171 | 0.531 | 2.586 |
| Drinking volume | -0.008 | 0.992 | 0.019 | -0.469 | 0.639 | 0.992 | 0.961 | 1.025 |
| Smoke | 0.004 | 1.004 | 0.102 | 0.044 | 0.965 | 1.004 | 0.844 | 1.194 |
| Diet |  |  |  |  |  |  |  |  |
| Fruit | -0.046 | 0.956 | 0.045 | -1.072 | 0.284 | 0.956 | 0.879 | 1.038 |
| Veg | -0.094 | 0.910 | 0.054 | -1.857 | 0.063 | 0.910 | 0.824 | 1.005 |
| Fish | 0.181 | 1.198 | 0.064 | 3.105 | 0.002 | 1.198 | 1.069 | 1.343 |
| Legume | 0.213 | 1.238 | 0.060 | 3.698 | 0.000 | 1.238 | 1.105 | 1.386 |
| Sugar | -0.144 | 0.866 | 0.054 | -2.862 | 0.004 | 0.866 | 0.784 | 0.956 |
| Activity |  |  |  |  |  |  |  |  |
| Housework | 0.065 | 1.067 | 0.052 | 1.317 | 0.188 | 1.067 | 0.969 | 1.174 |
| Outdoor | 0.047 | 1.048 | 0.050 | 0.983 | 0.325 | 1.048 | 0.954 | 1.152 |
| Gardening | 0.073 | 1.076 | 0.078 | 1.026 | 0.305 | 1.076 | 0.936 | 1.237 |
| Raise pet | -0.045 | 0.956 | 0.055 | -0.882 | 0.378 | 0.956 | 0.864 | 1.057 |
| Mahjong | 0.192 | 1.212 | 0.083 | 2.476 | 0.013 | 1.212 | 1.041 | 1.411 |

| **Table.S2** Results of dynamic Cox model IV (contains samples from the 2^nd^ random sample, n=1292) | | | | | | | | |
| --- | --- | --- | --- | --- | --- | --- | --- | --- |
| Factor | coef | se (coef) | robust se | *z* | *p* | *HR* | 95%*CI* for *HR* | |
|  |  |  |  |  |  |  | Lower bound | Upper bound |
| Age | 0.043 | 1.044 | 0.005 | 9.368 | 0.000 | 1.044 | 1.035 | 1.053 |
| Gender | 0.390 | 1.477 | 0.108 | 4.121 | 0.000 | 1.477 | 1.227 | 1.777 |
| Years of education | -0.009 | 0.991 | 0.014 | -0.702 | 0.482 | 0.991 | 0.968 | 1.016 |
| Drinking years | 0.004 | 1.004 | 0.002 | 1.976 | 0.048 | 1.004 | 1.000 | 1.008 |
| Category |  |  |  |  |  |  |  |  |
| Non-alcoholic | Reference | | | | | | | |
| Liquor (≥ 38º) | 0.731 | 2.077 | 0.162 | 4.965 | 0.000 | 2.077 | 1.556 | 2.772 |
| Liquor (< 38º) | 0.446 | 1.562 | 0.195 | 2.619 | 0.009 | 1.562 | 1.119 | 2.182 |
| Wine | 0.622 | 1.862 | 0.210 | 3.385 | 0.001 | 1.862 | 1.299 | 2.669 |
| Rice wine | 0.481 | 1.618 | 0.188 | 2.798 | 0.005 | 1.619 | 1.155 | 2.268 |
| Beer | 0.183 | 1.200 | 0.444 | 0.444 | 0.657 | 1.200 | 0.536 | 2.689 |
| Others | 0.172 | 1.187 | 0.471 | 0.420 | 0.674 | 1.187 | 0.533 | 2.645 |
| Drinking volume | -0.003 | 0.997 | 0.019 | -0.179 | 0.858 | 0.997 | 0.965 | 1.030 |
| Smoke | 0.089 | 1.093 | 0.100 | 1.053 | 0.292 | 1.093 | 0.926 | 1.291 |
| Diet |  |  |  |  |  |  |  |  |
| Fruit | -0.072 | 0.930 | 0.045 | -1.688 | 0.091 | 0.930 | 0.856 | 1.012 |
| Veg | -0.154 | 0.857 | 0.057 | -2.889 | 0.004 | 0.857 | 0.772 | 0.952 |
| Fish | 0.188 | 1.206 | 0.063 | 3.175 | 0.001 | 1.206 | 1.075 | 1.355 |
| Legume | 0.173 | 1.189 | 0.062 | 2.972 | 0.003 | 1.189 | 1.061 | 1.332 |
| Sugar | -0.146 | 0.864 | 0.055 | -2.864 | 0.004 | 0.864 | 0.782 | 0.955 |
| Activity |  |  |  |  |  |  |  |  |
| Housework | 0.080 | 1.083 | 0.053 | 1.647 | 0.100 | 1.084 | 0.985 | 1.192 |
| Outdoor | 0.053 | 1.055 | 0.051 | 1.071 | 0.284 | 1.055 | 0.957 | 1.162 |
| Gardening | 0.201 | 1.223 | 0.083 | 2.672 | 0.008 | 1.223 | 1.055 | 1.418 |
| Raise pet | -0.092 | 0.912 | 0.056 | -1.765 | 0.078 | 0.912 | 0.823 | 1.010 |
| Mahjong | 0.289 | 1.335 | 0.085 | 3.835 | 0.000 | 1.335 | 1.152 | 1.548 |

| **Table.S3** Results of dynamic Cox model IV (contains samples from the 3^rd^ random sample, n=1292) | | | | | | | | |
| --- | --- | --- | --- | --- | --- | --- | --- | --- |
| Factor | coef | se (coef) | robust se | *z* | *p* | *HR* | 95%*CI* for *HR* | |
|  |  |  |  |  |  |  | Lower bound | Upper bound |
| Age | 0.043 | 1.043 | 0.005 | 9.646 | 0.000 | 1.043 | 1.035 | 1.053 |
| Gender | 0.329 | 1.389 | 0.108 | 3.547 | 0.000 | 1.389 | 1.159 | 1.666 |
| Years of education | -0.010 | 0.990 | 0.014 | -0.786 | 0.432 | 0.990 | 0.966 | 1.015 |
| Drinking years | 0.004 | 1.004 | 0.002 | 2.079 | 0.038 | 1.004 | 1.000 | 1.008 |
| Category |  |  |  |  |  |  |  |  |
| Non-alcoholic | Reference | | | | | | | |
| Liquor (≥ 38º) | 0.705 | 2.023 | 0.161 | 4.908 | 0.000 | 2.023 | 1.527 | 2.680 |
| Liquor (< 38º) | 0.436 | 1.546 | 0.194 | 2.621 | 0.009 | 1.546 | 1.116 | 2.141 |
| Wine | 0.549 | 1.732 | 0.208 | 3.028 | 0.002 | 1.732 | 1.214 | 2.471 |
| Rice wine | 0.443 | 1.557 | 0.186 | 2.622 | 0.009 | 1.557 | 1.118 | 2.168 |
| Beer | 0.174 | 1.191 | 0.443 | 0.434 | 0.664 | 1.191 | 0.542 | 2.617 |
| Others | 0.108 | 1.114 | 0.471 | 0.261 | 0.794 | 1.114 | 0.495 | 2.506 |
| Drinking volume | -0.006 | 0.994 | 0.019 | -0.392 | 0.695 | 0.994 | 0.963 | 1.025 |
| Smoke | -0.107 | 0.898 | 0.103 | -1.230 | 0.219 | 0.898 | 0.757 | 1.066 |
| Diet |  |  |  |  |  |  |  |  |
| Fruit | -0.084 | 0.920 | 0.044 | -2.009 | 0.045 | 0.920 | 0.848 | 0.998 |
| Veg | -0.123 | 0.884 | 0.055 | -2.315 | 0.021 | 0.884 | 0.797 | 0.981 |
| Fish | 0.169 | 1.184 | 0.063 | 2.858 | 0.004 | 1.184 | 1.054 | 1.329 |
| Legume | 0.209 | 1.232 | 0.061 | 3.599 | 0.000 | 1.232 | 1.100 | 1.380 |
| Sugar | -0.123 | 0.884 | 0.054 | -2.471 | 0.013 | 0.884 | 0.802 | 0.975 |
| Activity |  |  |  |  |  |  |  |  |
| Housework | 0.107 | 1.113 | 0.052 | 2.187 | 0.029 | 1.113 | 1.011 | 1.225 |
| Outdoor | 0.097 | 1.101 | 0.051 | 1.952 | 0.051 | 1.102 | 1.000 | 1.214 |
| Gardening | 0.066 | 1.068 | 0.075 | 0.947 | 0.343 | 1.068 | 0.932 | 1.225 |
| Raise pet | -0.089 | 0.914 | 0.055 | -1.780 | 0.075 | 0.915 | 0.829 | 1.009 |
| Mahjong | 0.192 | 1.211 | 0.082 | 2.614 | 0.009 | 1.211 | 1.049 | 1.398 |

| **Table.S4** Results of dynamic Cox model IV (contains samples from the 4^th^ random sample, n=1292) | | | | | | | | |
| --- | --- | --- | --- | --- | --- | --- | --- | --- |
| Factor | coef | se (coef) | robust se | *z* | *p* | *HR* | 95%*CI* for *HR* | |
|  |  |  |  |  |  |  | Lower bound | Upper bound |
| Age | 0.043 | 1.044 | 0.005 | 9.571 | 0.000 | 1.044 | 1.035 | 1.053 |
| Gender | 0.397 | 1.487 | 0.111 | 4.180 | 0.000 | 1.487 | 1.235 | 1.792 |
| Years of education | -0.008 | 0.992 | 0.013 | -0.628 | 0.530 | 0.992 | 0.968 | 1.017 |
| Drinking years | 0.004 | 1.004 | 0.002 | 1.939 | 0.053 | 1.004 | 1.000 | 1.007 |
| Category |  |  |  |  |  |  |  |  |
| Non-alcoholic | Reference | | | | | | | |
| Liquor (≥ 38º) | 0.653 | 1.921 | 0.162 | 4.547 | 0.000 | 1.921 | 1.450 | 2.546 |
| Liquor (< 38º) | 0.390 | 1.478 | 0.196 | 2.311 | 0.021 | 1.478 | 1.061 | 2.058 |
| Wine | 0.526 | 1.693 | 0.210 | 2.867 | 0.004 | 1.693 | 1.181 | 2.426 |
| Rice wine | 0.409 | 1.506 | 0.187 | 2.430 | 0.015 | 1.506 | 1.082 | 2.096 |
| Beer | 0.124 | 1.132 | 0.443 | 0.311 | 0.756 | 1.133 | 0.517 | 2.482 |
| Others | 0.103 | 1.109 | 0.471 | 0.256 | 0.798 | 1.109 | 0.503 | 2.447 |
| Drinking volume | -0.005 | 0.995 | 0.019 | -0.292 | 0.770 | 0.995 | 0.964 | 1.028 |
| Smoke | 0.054 | 1.056 | 0.104 | 0.622 | 0.534 | 1.056 | 0.890 | 1.253 |
| Diet |  |  |  |  |  |  |  |  |
| Fruit | -0.081 | 0.922 | 0.045 | -1.905 | 0.057 | 0.922 | 0.848 | 1.002 |
| Veg | -0.119 | 0.887 | 0.055 | -2.297 | 0.022 | 0.888 | 0.802 | 0.983 |
| Fish | 0.256 | 1.291 | 0.065 | 4.255 | 0.000 | 1.291 | 1.148 | 1.453 |
| Legume | 0.159 | 1.172 | 0.062 | 2.627 | 0.009 | 1.172 | 1.041 | 1.320 |
| Sugar | -0.095 | 0.910 | 0.054 | -1.848 | 0.065 | 0.910 | 0.823 | 1.006 |
| Activity |  |  |  |  |  |  |  |  |
| Housework | 0.081 | 1.084 | 0.052 | 1.671 | 0.095 | 1.084 | 0.986 | 1.192 |
| Outdoor | 0.094 | 1.099 | 0.050 | 1.974 | 0.048 | 1.099 | 1.001 | 1.207 |
| Gardening | 0.064 | 1.066 | 0.075 | 0.945 | 0.345 | 1.066 | 0.934 | 1.217 |
| Raise pet | -0.071 | 0.932 | 0.054 | -1.398 | 0.162 | 0.932 | 0.844 | 1.029 |
| Mahjong | 0.201 | 1.223 | 0.081 | 2.729 | 0.006 | 1.223 | 1.058 | 1.413 |

| **Table.S5** Results of dynamic Cox model IV (contains samples from the 5^th^ random sample, n=1292) | | | | | | | | |
| --- | --- | --- | --- | --- | --- | --- | --- | --- |
| Factor | coef | se (coef) | robust se | *z* | *p* | *HR* | 95%*CI* for *HR* | |
|  |  |  |  |  |  |  | Lower bound | Upper bound |
| Age | 0.047 | 1.048 | 0.005 | 10.517 | 0.000 | 1.048 | 1.039 | 1.058 |
| Gender | 0.345 | 1.411 | 0.110 | 3.585 | 0.000 | 1.411 | 1.169 | 1.704 |
| Years of education | -0.019 | 0.981 | 0.014 | -1.525 | 0.127 | 0.981 | 0.957 | 1.006 |
| Drinking years | 0.004 | 1.004 | 0.002 | 1.849 | 0.064 | 1.004 | 1.000 | 1.007 |
| Category |  |  |  |  |  |  |  |  |
| Non-alcoholic | Reference | | | | | | | |
| Liquor (≥ 38º) | 0.641 | 1.899 | 0.161 | 4.462 | 0.000 | 1.899 | 1.433 | 2.516 |
| Liquor (< 38º) | 0.365 | 1.441 | 0.195 | 2.155 | 0.031 | 1.441 | 1.034 | 2.009 |
| Wine | 0.530 | 1.700 | 0.208 | 2.941 | 0.003 | 1.700 | 1.194 | 2.421 |
| Rice wine | 0.363 | 1.438 | 0.186 | 2.146 | 0.032 | 1.438 | 1.032 | 2.004 |
| Beer | 0.068 | 1.070 | 0.443 | 0.167 | 0.867 | 1.070 | 0.483 | 2.370 |
| Others | 0.073 | 1.076 | 0.470 | 0.180 | 0.857 | 1.076 | 0.486 | 2.381 |
| Drinking volume | -0.001 | 0.999 | 0.019 | -0.067 | 0.946 | 0.999 | 0.967 | 1.032 |
| Smoke | 0.065 | 1.068 | 0.103 | 0.735 | 0.462 | 1.068 | 0.897 | 1.272 |
| Diet |  |  |  |  |  |  |  |  |
| Fruit | -0.107 | 0.899 | 0.045 | -2.516 | 0.012 | 0.899 | 0.827 | 0.977 |
| Veg | -0.162 | 0.851 | 0.057 | -2.950 | 0.003 | 0.851 | 0.764 | 0.947 |
| Fish | 0.160 | 1.174 | 0.063 | 2.681 | 0.007 | 1.174 | 1.044 | 1.320 |
| Legume | 0.209 | 1.233 | 0.062 | 3.420 | 0.001 | 1.233 | 1.093 | 1.390 |
| Sugar | -0.147 | 0.863 | 0.054 | -2.932 | 0.003 | 0.864 | 0.783 | 0.953 |
| Activity |  |  |  |  |  |  |  |  |
| Housework | 0.043 | 1.044 | 0.053 | 0.856 | 0.392 | 1.044 | 0.946 | 1.151 |
| Outdoor | 0.050 | 1.052 | 0.051 | 1.031 | 0.303 | 1.052 | 0.956 | 1.158 |
| Gardening | 0.152 | 1.164 | 0.078 | 2.067 | 0.039 | 1.164 | 1.008 | 1.344 |
| Raise pet | -0.098 | 0.906 | 0.053 | -1.988 | 0.047 | 0.906 | 0.823 | 0.999 |
| Mahjong | 0.203 | 1.225 | 0.082 | 2.698 | 0.007 | 1.225 | 1.057 | 1.420 |

| **Table.S6** Results of dynamic Cox model IV (contains samples from the 6^th^ random sample, n=1292) | | | | | | | | |
| --- | --- | --- | --- | --- | --- | --- | --- | --- |
| Factor | coef | se (coef) | robust se | *z* | *p* | *HR* | 95%*CI* for *HR* | |
|  |  |  |  |  |  |  | Lower bound | Upper bound |
| Age | 0.042 | 1.043 | 0.005 | 9.133 | 0.000 | 1.043 | 1.034 | 1.053 |
| Gender | 0.305 | 1.357 | 0.108 | 3.272 | 0.001 | 1.357 | 1.130 | 1.629 |
| Years of education | -0.009 | 0.991 | 0.013 | -0.763 | 0.445 | 0.991 | 0.967 | 1.015 |
| Drinking years | 0.004 | 1.004 | 0.002 | 1.978 | 0.048 | 1.004 | 1.000 | 1.008 |
| Category |  |  |  |  |  |  |  |  |
| Non-alcoholic | Reference | | | | | | | |
| Liquor (≥ 38º) | 0.610 | 1.841 | 0.161 | 4.206 | 0.000 | 1.841 | 1.385 | 2.446 |
| Liquor (< 38º) | 0.349 | 1.417 | 0.194 | 2.105 | 0.035 | 1.417 | 1.024 | 1.960 |
| Wine | 0.508 | 1.661 | 0.209 | 2.768 | 0.006 | 1.661 | 1.160 | 2.380 |
| Rice wine | 0.370 | 1.448 | 0.187 | 2.176 | 0.030 | 1.448 | 1.037 | 2.021 |
| Beer | 0.078 | 1.082 | 0.443 | 0.199 | 0.843 | 1.082 | 0.498 | 2.347 |
| Others | 0.044 | 1.045 | 0.470 | 0.110 | 0.913 | 1.045 | 0.477 | 2.288 |
| Drinking volume | -0.005 | 0.995 | 0.019 | -0.294 | 0.769 | 0.995 | 0.964 | 1.027 |
| Smoke | -0.017 | 0.983 | 0.102 | -0.192 | 0.848 | 0.983 | 0.828 | 1.168 |
| Diet |  |  |  |  |  |  |  |  |
| Fruit | -0.036 | 0.965 | 0.044 | -0.853 | 0.394 | 0.965 | 0.889 | 1.047 |
| Veg | -0.152 | 0.859 | 0.056 | -2.843 | 0.004 | 0.859 | 0.774 | 0.954 |
| Fish | 0.181 | 1.199 | 0.063 | 3.071 | 0.002 | 1.199 | 1.068 | 1.346 |
| Legume | 0.140 | 1.151 | 0.059 | 2.458 | 0.014 | 1.151 | 1.029 | 1.287 |
| Sugar | -0.099 | 0.906 | 0.053 | -1.991 | 0.047 | 0.906 | 0.822 | 0.999 |
| Activity |  |  |  |  |  |  |  |  |
| Housework | 0.095 | 1.099 | 0.052 | 1.938 | 0.053 | 1.099 | 0.999 | 1.210 |
| Outdoor | 0.087 | 1.091 | 0.051 | 1.776 | 0.076 | 1.091 | 0.991 | 1.202 |
| Gardening | 0.096 | 1.101 | 0.076 | 1.368 | 0.171 | 1.101 | 0.959 | 1.263 |
| Raise pet | -0.080 | 0.923 | 0.054 | -1.542 | 0.123 | 0.923 | 0.834 | 1.022 |
| Mahjong | 0.105 | 1.110 | 0.079 | 1.462 | 0.144 | 1.110 | 0.965 | 1.278 |

| **Table.S7** Results of dynamic Cox model IV (contains samples from the 7^th^ random sample, n=1292) | | | | | | | | |
| --- | --- | --- | --- | --- | --- | --- | --- | --- |
| Factor | coef | se (coef) | robust se | *z* | *p* | *HR* | 95%*CI* for *HR* | |
|  |  |  |  |  |  |  | Lower bound | Upper bound |
| Age | 0.046 | 1.047 | 0.005 | 10.727 | 0.000 | 1.047 | 1.038 | 1.056 |
| Gender | 0.379 | 1.462 | 0.108 | 3.952 | 0.000 | 1.462 | 1.211 | 1.764 |
| Years of education | -0.017 | 0.984 | 0.013 | -1.347 | 0.178 | 0.984 | 0.960 | 1.008 |
| Drinking years | 0.004 | 1.004 | 0.002 | 1.865 | 0.062 | 1.004 | 1.000 | 1.007 |
| Category |  |  |  |  |  |  |  |  |
| Non-alcoholic | Reference | | | | | | | |
| Liquor (≥ 38º) | 0.542 | 1.720 | 0.159 | 3.679 | 0.000 | 1.720 | 1.288 | 2.296 |
| Liquor (< 38º) | 0.326 | 1.386 | 0.193 | 1.947 | 0.051 | 1.386 | 0.998 | 1.925 |
| Wine | 0.464 | 1.590 | 0.208 | 2.522 | 0.012 | 1.590 | 1.109 | 2.281 |
| Rice wine | 0.293 | 1.340 | 0.185 | 1.711 | 0.087 | 1.340 | 0.958 | 1.875 |
| Beer | 0.056 | 1.058 | 0.443 | 0.137 | 0.891 | 1.058 | 0.474 | 2.360 |
| Others | 0.084 | 1.087 | 0.469 | 0.216 | 0.829 | 1.088 | 0.508 | 2.330 |
| Drinking volume | -0.005 | 0.995 | 0.019 | -0.310 | 0.756 | 0.995 | 0.962 | 1.029 |
| Smoke | 0.142 | 1.153 | 0.102 | 1.555 | 0.120 | 1.153 | 0.964 | 1.380 |
| Diet |  |  |  |  |  |  |  |  |
| Fruit | -0.032 | 0.968 | 0.044 | -0.747 | 0.455 | 0.968 | 0.890 | 1.054 |
| Veg | -0.204 | 0.815 | 0.058 | -3.640 | 0.000 | 0.815 | 0.730 | 0.910 |
| Fish | 0.216 | 1.241 | 0.062 | 3.703 | 0.000 | 1.241 | 1.107 | 1.392 |
| Legume | 0.154 | 1.167 | 0.061 | 2.558 | 0.011 | 1.167 | 1.037 | 1.313 |
| Sugar | -0.191 | 0.826 | 0.053 | -3.772 | 0.000 | 0.826 | 0.748 | 0.912 |
| Activity |  |  |  |  |  |  |  |  |
| Housework | 0.034 | 1.035 | 0.051 | 0.702 | 0.483 | 1.035 | 0.941 | 1.138 |
| Outdoor | 0.127 | 1.135 | 0.050 | 2.549 | 0.011 | 1.135 | 1.030 | 1.252 |
| Gardening | 0.097 | 1.102 | 0.074 | 1.406 | 0.160 | 1.102 | 0.963 | 1.261 |
| Raise pet | -0.108 | 0.898 | 0.053 | -2.155 | 0.031 | 0.898 | 0.814 | 0.990 |
| Mahjong | 0.226 | 1.254 | 0.082 | 2.962 | 0.003 | 1.254 | 1.080 | 1.457 |

| **Table.S8** Results of dynamic Cox model IV (contains samples from the 8^th^ random sample, n=1292) | | | | | | | | |
| --- | --- | --- | --- | --- | --- | --- | --- | --- |
| Factor | coef | se (coef) | robust se | *z* | *p* | *HR* | 95%*CI* for *HR* | |
|  |  |  |  |  |  |  | Lower bound | Upper bound |
| Age | 0.041 | 1.042 | 0.005 | 9.088 | 0.000 | 1.042 | 1.033 | 1.051 |
| Gender | 0.372 | 1.450 | 0.109 | 3.982 | 0.000 | 1.450 | 1.208 | 1.741 |
| Years of education | -0.009 | 0.991 | 0.014 | -0.685 | 0.493 | 0.991 | 0.967 | 1.017 |
| Drinking years | 0.004 | 1.004 | 0.002 | 1.969 | 0.049 | 1.004 | 1.000 | 1.008 |
| Category |  |  |  |  |  |  |  |  |
| Non-alcoholic | Reference | | | | | | | |
| Liquor (≥ 38º) | 0.652 | 1.919 | 0.162 | 4.454 | 0.000 | 1.919 | 1.441 | 2.557 |
| Liquor (< 38º) | 0.403 | 1.497 | 0.194 | 2.405 | 0.016 | 1.497 | 1.078 | 2.080 |
| Wine | 0.546 | 1.726 | 0.209 | 3.013 | 0.003 | 1.727 | 1.210 | 2.463 |
| Rice wine | 0.406 | 1.502 | 0.186 | 2.418 | 0.016 | 1.502 | 1.080 | 2.088 |
| Beer | 0.103 | 1.109 | 0.442 | 0.259 | 0.796 | 1.109 | 0.508 | 2.423 |
| Others | 0.130 | 1.138 | 0.470 | 0.324 | 0.746 | 1.138 | 0.519 | 2.497 |
| Drinking volume | -0.004 | 0.996 | 0.018 | -0.245 | 0.806 | 0.996 | 0.966 | 1.027 |
| Smoke | 0.052 | 1.053 | 0.103 | 0.591 | 0.555 | 1.054 | 0.886 | 1.252 |
| Diet |  |  |  |  |  |  |  |  |
| Fruit | -0.088 | 0.915 | 0.046 | -2.047 | 0.041 | 0.915 | 0.841 | 0.996 |
| Veg | -0.038 | 0.963 | 0.053 | -0.758 | 0.449 | 0.963 | 0.874 | 1.062 |
| Fish | 0.163 | 1.177 | 0.063 | 2.794 | 0.005 | 1.177 | 1.050 | 1.320 |
| Legume | 0.155 | 1.167 | 0.061 | 2.636 | 0.008 | 1.167 | 1.040 | 1.310 |
| Sugar | -0.106 | 0.900 | 0.054 | -2.056 | 0.040 | 0.900 | 0.814 | 0.995 |
| Activity |  |  |  |  |  |  |  |  |
| Housework | 0.060 | 1.062 | 0.052 | 1.259 | 0.208 | 1.062 | 0.967 | 1.166 |
| Outdoor | 0.108 | 1.114 | 0.050 | 2.186 | 0.029 | 1.114 | 1.011 | 1.227 |
| Gardening | 0.106 | 1.112 | 0.079 | 1.427 | 0.153 | 1.112 | 0.961 | 1.287 |
| Raise pet | -0.060 | 0.942 | 0.055 | -1.183 | 0.237 | 0.942 | 0.853 | 1.040 |
| Mahjong | 0.180 | 1.198 | 0.075 | 2.681 | 0.007 | 1.198 | 1.050 | 1.367 |

| **Table.S9** Results of dynamic Cox model IV (contains samples from the 9^th^ random sample, n=1292) | | | | | | | | |
| --- | --- | --- | --- | --- | --- | --- | --- | --- |
| Factor | coef | se (coef) | robust se | *z* | *p* | *HR* | 95%*CI* for *HR* | |
|  |  |  |  |  |  |  | Lower bound | Upper bound |
| Age | 0.041 | 1.042 | 0.005 | 9.287 | 0.000 | 1.042 | 1.033 | 1.051 |
| Gender | 0.394 | 1.483 | 0.110 | 4.167 | 0.000 | 1.483 | 1.232 | 1.785 |
| Years of education | -0.013 | 0.987 | 0.013 | -1.048 | 0.295 | 0.987 | 0.963 | 1.012 |
| Drinking years | 0.004 | 1.004 | 0.002 | 1.950 | 0.051 | 1.004 | 1.000 | 1.007 |
| Category |  |  |  |  |  |  |  |  |
| Non-alcoholic | Reference | | | | | | | |
| Liquor (≥ 38º) | 0.578 | 1.783 | 0.161 | 4.005 | 0.000 | 1.783 | 1.343 | 2.366 |
| Liquor (< 38º) | 0.333 | 1.396 | 0.194 | 2.008 | 0.045 | 1.396 | 1.008 | 1.933 |
| Wine | 0.472 | 1.604 | 0.209 | 2.598 | 0.009 | 1.604 | 1.123 | 2.290 |
| Rice wine | 0.348 | 1.417 | 0.187 | 2.073 | 0.038 | 1.417 | 1.019 | 1.969 |
| Beer | 0.040 | 1.041 | 0.443 | 0.101 | 0.920 | 1.041 | 0.474 | 2.286 |
| Others | 0.075 | 1.078 | 0.470 | 0.190 | 0.849 | 1.078 | 0.497 | 2.337 |
| Drinking volume | -0.004 | 0.996 | 0.019 | -0.228 | 0.819 | 0.996 | 0.965 | 1.029 |
| Smoke | 0.094 | 1.098 | 0.103 | 1.044 | 0.296 | 1.098 | 0.921 | 1.309 |
| Diet |  |  |  |  |  |  |  |  |
| Fruit | -0.071 | 0.932 | 0.044 | -1.714 | 0.087 | 0.932 | 0.860 | 1.010 |
| Veg | -0.144 | 0.866 | 0.055 | -2.742 | 0.006 | 0.866 | 0.781 | 0.960 |
| Fish | 0.182 | 1.200 | 0.062 | 3.191 | 0.001 | 1.200 | 1.073 | 1.342 |
| Legume | 0.154 | 1.166 | 0.060 | 2.610 | 0.009 | 1.166 | 1.039 | 1.309 |
| Sugar | -0.150 | 0.861 | 0.053 | -3.077 | 0.002 | 0.861 | 0.782 | 0.947 |
| Activity |  |  |  |  |  |  |  |  |
| Housework | 0.045 | 1.046 | 0.052 | 0.925 | 0.355 | 1.046 | 0.951 | 1.150 |
| Outdoor | 0.099 | 1.104 | 0.051 | 1.993 | 0.046 | 1.104 | 1.002 | 1.216 |
| Gardening | 0.142 | 1.152 | 0.076 | 2.020 | 0.043 | 1.152 | 1.004 | 1.322 |
| Raise pet | -0.062 | 0.940 | 0.054 | -1.212 | 0.226 | 0.940 | 0.850 | 1.039 |
| Mahjong | 0.173 | 1.189 | 0.081 | 2.275 | 0.023 | 1.189 | 1.024 | 1.380 |

| **Table.S10** Results of dynamic Cox model IV (contains samples from the 10^th^ random sample, n=1292) | | | | | | | | |
| --- | --- | --- | --- | --- | --- | --- | --- | --- |
| Factor | coef | se (coef) | robust se | *z* | *p* | *HR* | 95%*CI* for *HR* | |
|  |  |  |  |  |  |  | Lower bound | Upper bound |
| Age | 0.042 | 1.043 | 0.005 | 9.432 | 0.000 | 1.043 | 1.034 | 1.052 |
| Gender | 0.360 | 1.433 | 0.110 | 3.772 | 0.000 | 1.433 | 1.189 | 1.728 |
| Years of education | -0.011 | 0.989 | 0.013 | -0.908 | 0.364 | 0.989 | 0.966 | 1.013 |
| Drinking years | 0.004 | 1.004 | 0.002 | 2.075 | 0.038 | 1.004 | 1.000 | 1.008 |
| Category |  |  |  |  |  |  |  |  |
| Non-alcoholic | Reference | | | | | | | |
| Liquor (≥ 38º) | 0.640 | 1.897 | 0.161 | 4.326 | 0.000 | 1.897 | 1.419 | 2.535 |
| Liquor (< 38º) | 0.380 | 1.462 | 0.193 | 2.242 | 0.025 | 1.462 | 1.049 | 2.038 |
| Wine | 0.573 | 1.773 | 0.208 | 3.115 | 0.002 | 1.773 | 1.237 | 2.542 |
| Rice wine | 0.447 | 1.564 | 0.186 | 2.656 | 0.008 | 1.564 | 1.124 | 2.175 |
| Beer | 0.138 | 1.149 | 0.444 | 0.343 | 0.732 | 1.149 | 0.520 | 2.535 |
| Others | 0.118 | 1.125 | 0.470 | 0.292 | 0.771 | 1.125 | 0.511 | 2.478 |
| Drinking volume | -0.013 | 0.987 | 0.019 | -0.743 | 0.458 | 0.987 | 0.955 | 1.021 |
| Smoke | 0.069 | 1.071 | 0.106 | 0.755 | 0.451 | 1.071 | 0.896 | 1.281 |
| Diet |  |  |  |  |  |  |  |  |
| Fruit | -0.031 | 0.969 | 0.045 | -0.732 | 0.464 | 0.969 | 0.891 | 1.054 |
| Veg | -0.147 | 0.863 | 0.057 | -2.662 | 0.008 | 0.863 | 0.775 | 0.962 |
| Fish | 0.157 | 1.170 | 0.064 | 2.589 | 0.010 | 1.170 | 1.039 | 1.317 |
| Legume | 0.129 | 1.138 | 0.061 | 2.157 | 0.031 | 1.138 | 1.012 | 1.280 |
| Sugar | -0.163 | 0.849 | 0.053 | -3.320 | 0.001 | 0.850 | 0.772 | 0.935 |
| Activity |  |  |  |  |  |  |  |  |
| Housework | 0.125 | 1.133 | 0.052 | 2.534 | 0.011 | 1.133 | 1.029 | 1.248 |
| Outdoor | 0.089 | 1.093 | 0.051 | 1.799 | 0.072 | 1.093 | 0.992 | 1.204 |
| Gardening | 0.008 | 1.008 | 0.074 | 0.114 | 0.910 | 1.008 | 0.879 | 1.155 |
| Raise pet | -0.050 | 0.952 | 0.055 | -0.962 | 0.336 | 0.952 | 0.860 | 1.053 |
| Mahjong | 0.171 | 1.187 | 0.081 | 2.226 | 0.026 | 1.187 | 1.021 | 1.380 |
